# Managerial workarounds in three European DRG systems

**DOI:** 10.1108/JHOM-10-2019-0295

**Published:** 2020-02-08

**Authors:** Rod Sheaff, Verdiana Morando, Naomi Chambers, Mark Exworthy, Ann Mahon, Richard Byng, Russell Mannion

**Affiliations:** 1 School of Law, Criminology and Government, Plymouth University, Plymouth, UK; 2 CERGAS Research Centre, SDA Bocconi Scuola di Direzione Aziendale, Milano, Lombardia, Italy; 3 GSD Healthcare, Dubai, United Arab Emirates; 4 Alliance Manchester Business School, The University of Manchester, Manchester, UK; 5 Plymouth University Peninsula Schools of Medicine and Dentistry, Plymouth University, Plymouth, UK; 6 University of Birmingham, Birmingham, UK

**Keywords:** Germany, Italy, England, DRG, Diagnostic related group, Managerial workaround

## Abstract

**Purpose:**

Attempts to transform health systems have in many countries involved starting to pay healthcare providers through a DRG system, but that has involved managerial workarounds. Managerial workarounds have seldom been analysed. This paper does so by extending and modifying existing knowledge of the causes and character of clinical and IT workarounds, to produce a conceptualisation of the managerial workaround. It further develops and revises this conceptualisation by comparing the practical management, at both provider and purchaser levels, of hospital DRG payment systems in England, Germany and Italy.

**Design/methodology/approach:**

We make a qualitative test of our initial assumptions about the antecedents, character and consequences of managerial workarounds by comparing them with a systematic comparison of case studies of the DRG hospital payment systems in England, Germany and Italy. The data collection through key informant interviews ( *N* = 154), analysis of policy documents ( *N* = 111) and an action learning set, began in 2010–12, with additional data collection from key informants and administrative documents continuing in 2018–19 to supplement and update our findings.

**Findings:**

Managers in all three countries developed very similar workarounds to contain healthcare costs to payers. To weaken DRG incentives to increase hospital activity, managers agreed to lower DRG payments for episodes of care above an agreed case-load ‘ceiling' and reduced payments by less than the full DRG amounts when activity fell below an agreed ‘floor' volume.

**Research limitations/implications:**

Empirically this study is limited to three OECD health systems, but since our findings come from both Bismarckian (social-insurance) and Beveridge (tax-financed) systems, they are likely to be more widely applicable. In many countries, DRGs coexist with non-DRG or pre-DRG systems, so these findings may also reflect a specific, perhaps transient, stage in DRG-system development. Probably there are also other kinds of managerial workaround, yet to be researched. Doing so would doubtlessly refine and nuance the conceptualisation of the ‘managerial workaround’ still further.

**Practical implications:**

In the case of DRGs, the managerial workarounds were instances of ‘constructive deviance' which enabled payers to reduce the adverse financial consequences, for them, arising from DRG incentives. The understanding of apparent failures or part-failures to transform a health system can be made more nuanced, balanced and diagnostic by using the concept of the ‘managerial workaround'.

**Social implications:**

Managerial workarounds also appear outside the health sector, so the present analysis of managerial workarounds may also have application to understanding attempts to transform such sectors as education, social care and environmental protection.

**Originality/value:**

So far as we are aware, no other study presents and tests the concept of a ‘managerial workaround'. Pervasive, non-trivial managerial workarounds may be symptoms of mismatched policy objectives, or that existing health system structures cannot realise current policy objectives; but the workarounds themselves may also contain solutions to these problems.

Many attempts to increase healthcare providers' efficiency and contain the growth of costs have included a policy of paying healthcare providers through a Diagnostic Related Group (DRG) system. In practice that has involved managerial workarounds at the payer–provider interface. Unlike clinical and IT workarounds, managerial workarounds at organisational and inter-organisational level have seldom been analysed. This paper's original contribution is to extend the concept of workaround for application to managerial activities. It exposes some implications for health policy, including the transformation of health systems by means of DRG payment systems. First we abstract the generic characteristics, antecedents and consequences attributed to workarounds at work-process level. Next we infer what the corresponding characteristics, antecedents and consequences would be, for managerial activity. We then apply that conceptualisation empirically to analyse data about DRG payment systems for hospitals in England, Germany and Italy. The policy rationales and consequences of these three DRG systems are more widely reported than the managerial workarounds which help them operate, so we also add that analysis to the empirical literature. In light of our empirical findings, we refine and adjust our initial conceptualisation of managerial workarounds. We conclude that research into health system transformation, policy conflicts and implementation deficits can be made more nuanced and diagnostic by adding the concept of the ‘managerial workaround’ to the analytic and diagnostic repertoire.

## 1. Background

### 1.1 The prototype: work-process workarounds

Workarounds are the ways in which individual workers or work groups informally by-pass or alter the ways in which a formalised work process is executed, so that they can fulfil its task in another way ( Halbesleben *et al.* , 2008 ). Studies of clinical and healthcare IT workarounds have mostly focused on the informal modification, even subversion, of officially sanctioned work processes ( De Bono *et al.* , 2010 ; Halbesleben *et al.* , 2008 ), for example, in nursing ( De Bono *et al.* , 2013 ), operating theatre safety ( Reason, 2005 ), or the use of electronic patient records ( Bar-Lev, 2015 ). Work processes are anyway typically discretionary: within limits, workers adjust them according to circumstances. Workarounds, however, adjust them further, beyond officially prescribed boundaries, to create informal, unauthorised improvisations that replace official rules and work processes with alternatives that the improviser thinks are more effective or practicable. Often workarounds emerge as improvised repairs of ill-designed, incomplete, impracticable, over-restrictive or otherwise dysfunctional work processes ( Bar-Lev, 2015 ; Vogelsmeier *et al.* , 2008 ) which make complex tasks still more difficult ( De Bono *et al.* , 2013 ). Generally workarounds are responses to organisational problems ( Lalley and Malloch, 2010 ) (e.g. inter-professional or inter-departmental boundaries, role ambiguity), practical inadequacies of new technologies ( Coiera, 2007 ), over-work ( Guédon *et al.* , 2017 ), inflexible rules or communication blockages ( De Bono *et al.* , 2013 ).

Such workarounds therefore have four main characteristics. They firstly extend a work process (e.g. using paper alongside electronic records ( Ellingsen *et al.* , 2013 )) or partly transfer it to another profession ( Reiz and Gewald, 2016 ). Such extensions may then require non-standard use of the ‘boundary objects’ used to structure and coordinate work across professional and organisational boundaries (e.g. adding free-text notes to checklists) ( Bar-Lev, 2015 ), stretching discretion or exploiting under-definitions of work routines (e.g. making observations instead of asking the patient about her pain ( Wallenburg *et al.* , 2019 )), re-sequencing tasks (e.g. recording a patient as having taken medication before she took it) ( Koppel *et al.* , 2008 ), or using resources intended for one work process for another (e.g. ‘off-label’ prescribing: using a medicine to treat conditions for which it is not licensed). Clinicians may employ their own, alternative diagnostic typologies, recording a more acceptable or lucrative diagnosis on official paperwork and negotiating diagnoses with patients ( Whooley, 2010 ). These extensions enable staff to use a work process more widely than managers intended or for additional purposes (e.g. prescribing a placebo).

Secondly, other parts of a work process may either be used less than officially intended or simply ignored, for example, by disabling warning or safety equipment or disregarding its warnings ( Vogelsmeier *et al.* , 2008 ). Staff may omit elements ( Halbesleben *et al.* , 2008 ; Koppel *et al.* , 2008 ) that appear unnecessarily laborious (e.g. using voice-recorders instead of typing ( Reiz and Gewald, 2016 )).

Workarounds are, thirdly, organised informally. Some are enacted individually and others collectively, whether by tacit agreement ( De Bono *et al.* , 2013 ) or explicit negotiation ( Ellingsen *et al.* , 2013 ), for example, to defend professional ‘autonomy'. When managers do not just ignore, or even promote ( Cresswell *et al.* , 2016 ), them workarounds usually incur lower penalties for a worker than overt resistance will.

Fourth, the purpose of workarounds is to overcome the aforementioned problems so that the work process achieves its aims more fully and reliably. Some writers describe workarounds as ‘violations' ( Reason, 2005 ), ‘resistance’ ( Reiz and Gewald, 2016 ) or circumventing the systems for maintaining service quality and safety ( Guédon *et al.* , 2017 ), as error-creating ( Koppel *et al.* , 2008 ) or as obstacles to systematic problem-solving. Far from instantiating error, laziness, self-interest, neglect or incompetence, however, many workarounds are ‘reinventions’ ( Barrett and Stephens, 2017 ) which improve work-process resilience ( Alper and Karsh, 2009 ), even at some cost for the improvisers themselves, and if one workaround then necessitates others ( Ellingsen *et al.* , 2013 ). Kobayashi *et al.* (2005) it suggests that workarounds are temporary, but those which introduce demonstrably better work processes may gradually become normal working practice ( Vogelsmeier *et al.* , 2008 ; Zhou *et al.* , 2011 ), because they get the work done ( De Bono *et al.* , 2013 ).

### 1.2 Managerial workarounds

Managerial routines are above all the second-order routines through which managers coordinate, control, resource and monitor the activities of those who execute the technical, first-order work processes discussed earlier. To do so, managers typically use tailored combinations of persuasion, financial or other incentives, resource allocation, delegating responsibility, relationality, coercion and work monitoring. Managerial workarounds would affect the work of implementing policy mandates, whether national policy or organisational-level priorities, rather than clinical work, and affect organisational- and inter-organisational information flows. A repeat search (28th November 2019) of Google Scholar for English-language journal papers published during 2009–2019 which contained the phrase ‘managerial workaround’ or ‘management workaround’ (or ‘work-around’) still yielded just 15 hits, eight concerning IT only, four work-processes only, one accounting systems only and two concerning household or family firm settings: too little material for a systematic review. We had to develop the concept of a managerial workaround almost from scratch.

Transposing the earlier analysis of work-process workarounds onto managerial activity, one would expect managerial workarounds to occur when managers perceived the mandate as:

1. Relying on missing or unreliable organisational structures or systems, for example, for information sharing, staff deployment or procurement.
2. Facing organisational obstacles or resistance.
3. Having to be implemented through, or despite, restrictive regulations.
4. Threatening managers' occupational interests (e.g. power, income, status).
5. Involving conflicting objectives and incentives, so that implementing one policy obstructs another.
6. Involving difficult, complex tasks depending on factors that the managers cannot fully control or predict.

Correspondingly one would predict that managerial workarounds would extend formal organisational or inter-organisational structures and managerial practices, using them for additional purposes than mandated. Then, managers would use their managerial discretion maximally, use resources intended for one use also for another and adjust what organisational-level monitoring systems report and how managers responded. Inter-organisational relationships, co-ordination mechanisms and boundary objects would be similarly altered and re-used. Concomitantly the use of other organisational or inter-organisational structures and managerial practices might diminish. Managers would not implement (or only symbolically implement) what they regarded as laborious, redundant or perverse elements of a mandate. Like technical workarounds, managerial ones would be initiated and established informally, by tacit agreement or explicit negotiation, whether enacted individually or collectively.

Such managerial workarounds would serve the purpose of increasing the practical resilience of existing organisational and inter-organisational structures and of enhancing policy implementation. Their character is therefore likely also to reflect their particular organisational, health system and policy context. Work-process workarounds apply only to practitioners' activities within provider organisations, but managerial workarounds develop as much in payer as provider organisations. Since work-process workarounds are claimed to frustrate policy-driven attempts to make healthcare safer and of higher quality, the question arises of whether managerial workarounds, analogously, frustrate policy attempts to transform health systems.

### 1.3 DRG systems: incubator for managerial workarounds?

In the 1990s, many European health systems were being transformed into more market-like structures to make them more manageable, contain the growth of healthcare spending, promote competition and open healthcare provision to a wider range of providers (corporate, not-for-profit, owner-managed, etc.). One consequence was to adopt DRGs as the main pricing unit for hospital services. Each of the DRG groups together clinical diagnoses (usually from the International Classification of Diseases ) on the basis first of broad clinical speciality (‘Major Diagnostic Category'), then whether surgical or non-surgical treatment is usual and then by other characteristics, varying by country, which predict the total cost of care for the patients so diagnosed (e.g. length of stay). Each DRG is thus approximately homogeneous in terms of treatment costs within that country ( Busse *et al.* , 2013 ). DRGs thereby commodify health care in the sense of standardising the definitions of care groups and setting one standard price for each. We define a DRG system as the set of managerial arrangements and organisational structures that produces these groupings, allocates a corresponding price (‘tariff’) to each and contracts, monitors and pays healthcare providers accordingly. DRG systems have at least four characteristics which, the aforementioned suggests, are likely to necessitate managerial workarounds.

First, policymakers in several countries (e.g. England, Germany, the Netherlands, Greece) have tried to design DRG systems to achieve two somewhat conflicting aims. One was to make the costs of health care to payers more predictable and manageable ( Covaleski *et al.* , 1993 ), make providers more accountable to payers and prevent over-charging ( Polyzos *et al.* , 2013 ) by setting payment tariffs prospectively instead of reimbursing providers' actual costs retrospectively. However, DRG systems also incentivised providers to increase the number of cases treated and treatment intensity. Evidence from Europe and Australia, but less so from the United States (where DRGs replaced fee-for-service payments which were already an incentive to increase activity), tended to indicate increased hospital activity volumes and/or intensity following DRG introduction ( Krabbe-Alkemade *et al.* , 2017 ; Street *et al.* , 2011 ). Expanding healthcare budgets relieved the tension between cost control and incentives for provider activity but the 2008 financial crisis exacerbated it.

DRG systems secondly allowed providers scope for flexibility and innovation by setting a cost ceiling beneath which providers were free to reduce their costs, whether by reducing capital intensity, using less labour, de-skilling, adopting new models of care, selecting patients or shifting costs elsewhere (e.g. to patients, when reducing lengths of stay) ( Schreyögg *et al.* , 2006 ). (To stop hospitals reducing lengths of stay too much, many DRG systems pay the hospital nothing more if a patient is re-admitted for treatment for the same condition too soon after discharge (30 days in England and Germany)). Nevertheless, differential innovation is likely to make health system behaviour less predictable overall, and a guaranteed DRG payment means that any savings all accrue to providers not payers.

With other concurrent healthcare reforms a DRG system tended, and in some European countries was intended, to promote competition. On the payer side, DRGs were originally designed for subscriber-based healthcare insurance markets or quasi-markets, including those where a third party (government, social health insurer (SHI), employer or corporate insurer) often pays on the patient's behalf. The reforms which promoted DRG systems were also intended, in Germany and the Netherlands, to stimulate competition between the SHIs. On the provider side, DRG systems were intended to stimulate yard-stick competition between providers (in England and Germany, regional and national comparisons of providers are the yard-sticks), on the basis of quality rather than cost. Whatever its benefits, competition might also be expected to make overall health system behaviour less stable, predictable and manageable.

Last, a DRG system is highly structured, being implemented through a complex, voluminous system of rules, regulations and calculations which must all be reviewed and updated regularly as clinical classifications, working practices, costs and technologies change.

Other possible antecedents of managerial workarounds do not so obviously apply to DRG systems. As noted, DRG systems tend to have extensive rather than missing regulations, organisational structures and information systems and if anything tend to support rather than threaten managers' occupational interests. Because DRG systems alter some of the demarcations between managers and clinicians, clinicians might be expected to resist them. Yet reports of such resistance to DRGs themselves are rare, partly, perhaps, because DRGs were designed to be intelligible and meaningful to clinicians. Nevertheless, sufficient antecedents do apply, to make it likely that DRG systems will incubate managerial workarounds. For example, studies already report providers up-coding patients to more lucrative tariffs (‘DRG creep') and supernumerary negotiations about outlier patients ( Cots *et al.* , 2011 ). If we wish to discover whether the concept of a managerial workaround has empirical application, DRG systems would be a good place to look.

## 2. Research questions

Using data about three DRG systems we therefore consider:

1. To what extent is the concept of ‘managerial workaround’ capable of empirical application, particularly to inter-organisational relationships between payers and providers?
  Insofar as it is applicable:
2. What are the characteristics of such managerial workarounds?
3. What antecedents or circumstances motivate them?
4. What does the concept of ‘managerial workaround’ imply for health policy analysis, hence policy formulation?

## 3. Methods

### 3.1 Research design

The study design was to make a qualitative test of the foregoing assumptions about the antecedents, character and consequences of managerial workarounds. The concept of a managerial workaround centres on the contrast between the official formulation of a policy mandate or local managerial priorities and the partly contrasting activities that its implementers undertake in practice. We elicited policymakers' stated intentions (summarised earlier) for their DRG systems from published policy documents and interviews with informants in the relevant national-level (England, Germany) and regional bodies (Italy). The researchers undertook this work in the respective languages. As evidence about how the system was implemented, and any workarounds, we use data from a larger study of how healthcare payers exercise governance over service providers (full research report obtainable from the authors).

### 3.2 Sampling

DRG systems have the same basic architecture but some technical and implementation details differ between health systems. Italy and England are Beveridge health systems which have adopted DRG payments to hospitals, Germany the paradigm Bismarckian health system to do so. German DRGs modify the Australian AR-DRG system. Italian DRGs followed the US (HCFA) model as did English HRGs (but less closely). By contrasting these systems, we aimed to elicit which practices, including workarounds, for implementing DRGs were specific to one particular health system and which were common to all three hence likely to reflect the nature of DRG systems per se .

Our sampling strategy was to select study sites which by the standards of their respective health systems had well-developed DRG systems. What that criterion meant in concrete terms differed for each country. In England, Clinical Commissioning Groups (CCG) ‘commission', that is, select, contract and pay providers. We selected early established ones as likely to have the most developed commissioning systems. (CCGs replaced the essentially similar Primary Care Trusts after 2013. In our study sites, their geographical configuration and membership had not changed.) For Germany, we selected the SHI whose DRG systems appeared to have most developed negotiating, informatics, monitoring and research capacity, and for Italy, the province making the most extensive use of DRGs. Our study sites were:

1. Four English CCGs which together covered the range of CCG organisational structures, of degree of local hospital competition (by Herfindahl–Hirschmann index) and extent of collaboration with neighbouring CCGs.
2. A large German SHI covering the whole country (over 7 million members) and having over 100 years' experience as a payer.
3. Lombardy health region, the first (1995), and when we started fieldwork only, Italian region using DRGs as its main hospital payment system.

### 3.3 Data collection

We used similar data collection methods in each country, but (as the funder required) more extensively in England. In each site we assembled a sample of key informants by snowballing from the most senior commissioning managers, who identified their counterpart lead clinicians and managers in secondary care providers and, as applicable, other organisations involved in the payment system (e.g. at national and/or regional government levels). Since workarounds involve adapting and supplementing official policies, rules and working processes, they were more likely to be reported in interviews, media rapportage and the action learning sets which ran in parallel with this research (see further discussion) than in official policy documents. Official documents and press rapportage, however, offer the most direct account of the DRG system's policy rationale. We conducted key informant interviews (20 in Germany, 24 in Italy, 110 in England) of managers using DRGs at provider ( *N* = 45) and payer ( *N* = 109) levels, content-analysed key policy and guidance documents (39 in Germany, 14 in Italy, 57 in England; and for one German hospital, an anonymised set of their standard contract documents), press rapportage and published papers. Our informants identified for us the policy and guidance documents most relevant to them, and some published studies and press rapportage. We found further published papers and rapportage by keyword searches of on-line databases (e.g. PubMed) and reference-chasing from grey studies (e.g. WHO country reports). There were two main rounds of data collection, the first and larger in 2010–13 with a second round of supplementary data collection from key informants and administrative documents during 2018–19 to re-verify, update and extend our data and findings about all three DRG systems.

We ran an action learning set (in effect, a focus group of practitioners) in which informants from the three countries exchanged information and ideas about how they paid for health care in practice. One clinician and one manager from each of the English study sites participated in a preliminary set of five workshops. This led to a mini-conference in which informants from Germany and Italy joined the set to exchange updates and practical ideas. The participants agreed conclusions at each meeting and from the action learning set overall. Three of the researchers were facilitators and recorded the findings and conclusions on each occasion.

### 3.4 Analysis

To compare DRG systems systematically required a common analytic framework and therefore a framework analysis, assembling the data from all sources from all three countries. This was logically equivalent into tabulating the data with one (virtual) column per country and one (virtual) row per research question, followed by sub-analysing the findings for each research question into sub-themes. Juxtaposing data from the different sources for each country gave an immediate triangulation, exposing gaps, ambiguities or apparent contradictions in the data, thereby prompting additional data collection. So far as possible we analysed transcripts and texts directly in the original language, to avoid the mistake of re-framing findings from one system with concepts that only apply elsewhere (e.g. the term ‘commissioning’ has no precise German or Italian equivalent). The patterns in each virtual row answered our research questions, but also exposed where the inference of new categories or concepts was required to accommodate unforeseen empirical findings. That, indeed, was what suggested the distinction between IT or clinician-level workarounds and managerial workarounds, leading to inference of the further patterns reported next and which structure this paper as a whole.

### 3.5 Ethics

An NHS Research Ethics Committee gave ethical approval (reference 09/H0206/50) for fieldwork in England, the University of Plymouth for fieldwork elsewhere, subject to informants remaining anonymous.

## 4. Findings

Space limitations compel us mostly to narrate rather than quote the informants' data. Fuller details, including many quotations from interview audio-recordings, are however in the research report available from the authors.

### 4.1 System settings and policy problems

Although the German SHIs are largely self-financing (albeit with some government subsidy), recent health policy has emphasised cost containment. Federal bodies annually set workers' and employers' SHI contribution rates, which constrains the SHIs' global budget. The German DRG system reimburses the running costs of inpatient care but *Land* (province) governments remain responsible for hospital investment, hence each hospital's bed allocation. Unlike American but like English and Italian practice, DRGs include the cost of doctors' work. Every DRG has a point weighting. Each point earns a fixed number of Euros. The conversion rate differs *Land* by *Land* . Each hospital is entitled to payment for as many cases as it can attract. Generally the SHIs wish to avoid patient numbers, hence costs, rising uncontrollably and therefore require a way to mitigate the financial incentives that the DRG system gives hospitals ( Vogl, 2013 ) for case splitting (dividing up one episode into several, each with its own DRG), up-coding ( Jürges and Köberlein, 2015 ), increasing activity and even over-treatment. For example, an early study in cardiology, which accounts for about 10 percent of hospital spending, found that 56 percent of angioplasties were done in patients with stable, chronic angina, where angioplasty is not useful (indeed potentially harmful) ( Dissmann and de Ridder, 2002 ). As another example, hospitals can claim additional reimbursement if they can document nursing services additional to those covered by procedure code OPS 9-200 for extensive nursing care. The Medizinische Dienst der Krankenkassen, which verifies DRG payments to hospitals, reports widespread over-charging although our SHI informants said that this is usually due to mis-coding or mis-diagnosis; some hospitals mis-code nearly a third of cases but those that do then face delayed payment, even cash-flow problems.

In the Italian DRG system, regions are the main payer, with considerable regional variation in how they use DRGs. Even in the relatively wealthy region we studied, pressures for cost control have increased, and in 2019 it remains a national policy priority. Most Italian NHS services were reimbursed retrospectively through DRG-based tariffs, to reflect patient and GP choices of provider. As in Germany and the Netherlands ( Krabbe-Alkemade *et al.* , 2017 ), introduction of the DRG system was associated with a decrease in hospital admissions and length of stay, an increase in day-hospital admissions and greater severity of illness among hospitalised patients ( Louis *et al.* , 1999 ).

The English ‘Payment by Results' (PBR) system uses Health Resource Groups (HRGs). CCGs, nominally controlled by GPs, were the main payers. HRGs were defined independently of the US prototype but with an essentially DRG-like design and policy rationales and the aim of increasing provider activity so as to reduce hospital waiting lists, a politically sensitive issue in the United Kingdom. Our informants also reported that HRGs incentivised hospitals to treat more cases and more intensive treatments, although hospitals' rising marginal costs (e.g. for opening operating theatres at week-ends) limited the expansion. Our commissioner informants claimed that treatment thresholds had fallen and up-coding had appeared. Health policy pressures to contain NHS costs were strong in our English study sites. Combined with patients' rights to use ‘Any Qualified Provider', the HRG tariff system weakened the commissioners' power to control provider case load and case mix, hence overall costs.

Across all three countries, the DRG system posed a similar complex of problems. It incentivised hospitals to increase their volume and intensity of treatments (and ‘game’ the administration of payments) and to reduce the costs of each treatment, but that meant costs to the hospital not costs for the payers. Costs for the payers simply rose, the reverse of national policies for containing the overall growth of healthcare costs.

### 4.2 Extending the DRG system

Our German SHI and hospital manager informants described how SHIs work around the adverse incentive effects of the DRG system by negotiating collectively with each hospital, focusing on spreadsheets that predict the number and case mix of DRGs for the coming year, which implies DRG points allocation for the hospital. Hospitals' ‘medical control units’ calculate the costs and prepare the negotiations with the SHIs. In past years the negotiation had been based on historic case loads but had become increasingly guided by the SHIs' wishes to save costs, shape services regionally and contain service expansion. Around the agreed number of DRG points a ‘corridor' ( *Flur* ) was also agreed, defining an upper and a lower limit of the number of cases for each main group of DRGs. The parties also agreed what rebates the SHI will receive should the volume or case mix fall below that range and what payment the hospital would receive for justified additional work above it. These agreements imply notional budgets for each main area of hospital work. This practice gives a concrete, detailed way of modelling and managing hospital activity, case mix and revenue costs. It is possible to reduce or remove specific kinds of case, even whole departments, by reallocations within the total number of points, but the total tends to be an incremental increase on the previous year's figure, constrained within the ‘corridors’. Thus SHIs ‘bundle' DRG payments in large groups in the interest of cost-containment, while for hospitals DRG bundling forestalled external micro-scrutiny of their internal activities and gave a stable, predictable route to such increases in income as the payers could offer.

The Lombardy regional government's only lever of cost control through the DRG system was to vary DRG tariff rates and adjust the production ceiling stated in the contract with providers, that is, the levels of activity at which non-standard tariff payments are made. As cost-control pressures increased, ‘ceiling budgets’ were introduced into provider contracts, enforced by tariff caps should service use exceed the planned budget. For ambulatory and diagnostic services, a provider was guaranteed 95 percent of the previous year's expenditure and case load. For activity between 97 percent and 103 percent of the latter, the tariff was cut by 30 percent; for activity at 103 percent to 106 percent above the previous year the tariff was cut by 60 percent; and above that the tariff was zero. This arrangement incentivised providers to achieve the 106 percent level, so as to grow the next year's baseline budget. By setting a zero marginal tariff for case load above 106 percent of what had been agreed at the start of each commissioning cycle, the Lombardy commissioners contained costs by in effect constructing hybrid of cost-and-volume (below the 106 percent level) and global (‘block') contracts (above 106 percent). All Italian regions use ceiling budgets, although the ceiling levels vary.

In England, the study site commissioners worked around the incentive effects of HRGs by collecting tariff payments into blocks, creating what they called the ‘cap-and-collar’ or ‘managed PBR’ system. If provider activity fell below a certain ‘floor’ or (more likely) exceeded a certain ‘ceiling’ (in our London site, 5 percent above the expected case load), tariff payments for the marginal activity were reduced by an agreed amount, in one study site to zero. The only exception to this pattern was that one study commissioner changed its *de facto* global contract with a private hospital to HRG tariff payments, a movement in the opposite direction. Overall, though, the predominant response was to bundle tariff payments in order to dampen what were, for payers, perverse incentive effects. At national level, a shift towards lower, ‘best-practice’ tariff prices (based on costs in the most efficient, not average, hospitals) was announced. Hospital managers' reactions were mixed. They all entered into negotiations with payers, based on the ‘cap-and-collar’ workaround. However, in one outer London CCG, the main hospital agreed to a reduced volume of elective activity, but then still deliberately provided more services than agreed, presenting the CCG with a *fait accompli* and corresponding bill. Both in our study sites and elsewhere NHS hospitals tried (and sometimes managed) to move into and out of ‘cap-and-collar’ arrangements according to their short-term financial interests.

Health managers in all three systems thus independently invented almost exactly the same workaround to reduce DRGs' upward pressure on healthcare costs.

A second German workaround was selective or ‘rebate' contracts, which German SHIs have offered since 1998. As with some forms of American ‘managed care', patients who opt into these contracts have a restricted choice of providers in return for lower subscriptions. The selected providers get a lower DRG payment per case in return for a larger case load. Patients must opt into these and into integrated care programmes, but many patients assumed that only providers with difficulty attracting patients accepted such contracts. Where they did not have latitude to vary the tariff system, the Lombardy commissioners also tried to influence patients', and still more GPs', choice of hospital, including attempts to reduce cross-boundary patient flows, especially for highly specialized services (e.g. neurosurgery). (Cross-boundary flow was also an issue in Emilia Romagna.) For example, Mantova ASL, on the border with the Emilia Romagna and Veneto regions, introduced training schemes encouraging GPs to meet their patients' diagnostic and specialised ambulatory care needs within the region and created an incentive for hospitals to hire specialists to treat such patients. Although for planned treatments English NHS patients choose their hospital, the reality in our study sites (and most of the United Kingdom) was that the payers and GPs relied on just one hospital for about two-thirds of their secondary care, although for certain low- to medium-complexity planned treatments some corporate and not-for-profit hospitals also treated NHS patients under contract. However, non-local and non-NHS hospitals were by default paid the full DRG tariff so diverting patients there would not reduce costs for the payers. The patient-diversion workaround thus appeared in Germany and Italy but not in England.

### 4.3 Diminutions of the DRG system

Concomitantly, the study sites limited the commodification of care. As noted, payers and providers agreed to standard payments only for cases within the ‘corridor’ (see earlier discussion), with lower payments for those above or below.

Another way of de-commodifying DRG payments was to ‘bundle’ them. Patients with multiple, especially long-term, conditions often require multiple concurrent treatments, often from a network of different services. DRGs are designed for funding payments to a single provider, not networks of multiple providers. Some German SHIs therefore attempted to introduce disease-management programmes ( Kifmann, 2017 ) providing preventive case management and continuous care for certain chronic conditions (e.g. diabetes, COPD, depression, alcoholism). However,

‘this most of the time only takes place in very classical incidents such as knee, hips etc, that are of an orthopaedic nature. These are relatively easy to make and are all plannable operations that happen very often. For more complicated courses of treatment, there is much less' (SHI manager).

Some of these projects experimentally constructed inter-organisational care pathways linking primary and secondary care for certain patient groups, a few including many providers but most only two. Even then, because of the bilateral character of DRG payments, these networks required specially negotiated contracts rather than the usual DRG payments. Another workaround is also required from the provider side: to integrate the data about its patients, who collectively may subscribe to several different SHIs ( Hildebrandt *et al.* , 2010 ). In some of these *Integrierte Versorgung* (‘integrated care’) contracts, the DRG price limit for treatments was also waived. Indeed, a separate payment system was authorised for networks from 2012. However, as with DRGs these contracts had to be re-negotiated annually, causing high transaction costs for SHIs. Some 14,000 such schemes existed by 2010 but, because patients had to opt in, only about 5.5 percent of people were then enrolled in them, representing about 1 percent of healthcare spending. Patients appear somewhat sceptical of them ( Amelung *et al.* , 2012 ). Cost savings were claimed for 85 percent of the integrated care schemes ( Kielstra, 2011 ), but in Germany the SHIs also operate the sickness benefit scheme, which is where the savings would usually materialise.

English payers also wanted to work around the difficulty of paying the networks of providers which patients with mental health problems and other chronic conditions often require. CCGs did so by arranging for providers' representatives to meet in order to negotiate the assembly of coordinated care pathways, constituting a care network. They called this ‘micro-commissioning' and regarded it as a method of primary care integration. CCGs influenced these service providers primarily through negotiation and persuasion, not the payment system. One CCG engaged an American HMO to help its general practices redesign services for these care groups, building in risk assessment and telephone support services. Some CCGs outside our study sites experimentally used ‘year-of-care’ payments which in effect bundled the HRG payments for chronic disease into annual capitation payments.

In Lombardy, payments for these kinds of services were simply outside the DRG system altogether. Even for hospital care, a controversial workaround in Lombardy was to supplement DRGs with ‘Functions with no tariff’ ( *Funzioni non tariffate* : FNT) payments to reimburse activities that lacked DRG tariffs or that it was considered undesirable to limit access to (e.g. A&E services, dialysis, foetal and neonatal pathology). FNTs were variable and at Regional Authority discretion. It can be argued that they represented a system of risk-adjustment payments for corporate and non-profit hospitals that could not access other public funds. FNTs were also used for ex-post funding, typically to help public hospitals that had exceeded their budgets. However, the unclear legal status of FNTs had also created gaming problems so from 2015 the Regional Authority began to replace them by supplementing the original DRG system with further new DRGs of its own: a new workaround to reduce gaming and grey areas and make the payment system more consistent across private, public and non-for-profit providers. The 2015 reforms considerably changed regional health system governance and regulation, but nevertheless left the DRG system, and its workarounds, still working as described earlier. In Germany too, annual contract negotiations had to cover payments for new treatments not yet in the DRG system.

In contrast to some clinical or IT workarounds, the DRG systems in the countries we studied had little obvious impact, good or bad, on the quality of care. German DRGs give the SHIs few direct financial incentives to offer hospitals for improving the quality of care, which the aforementioned negotiations do not cover. Rather, quality of care is managed when new types of treatment are considered for inclusion in the DRG payment scheme. The Gemeine Bundesausschuss (national negotiating body for health policy) sets out quality norms and guidelines and approves new treatments or diagnostic procedures for temporary, interim payments while they are being evaluated. Those whose evaluation is positive become eligible for reimbursement through a DRG. In 2012 the Lombardy regional government paid a variable premium (adjustment) of plus or minus 2 percent of the budget to providers according to their performance against certain quality standards, but this was a limited project, directly controlled by the regional general directorates. The English HRG system contained no direct incentive for improving or reducing service quality, although in one of our study sites, the district hospitals wanted to discontinue out-posted clinics in small rural community hospitals because HRG payments did not cover the cost, even though the commissioner valued these services. When tariff levels exceeded the cost of providing hospital services, though, HRGs incentivised hospitals not to transfer services to primary care. In all three systems, our informants did not describe any workarounds to the DRG system for the purposes of managing the quality of care.

### 4.4 Informal organisation

In all three countries, these workarounds were formulated during the contract negotiations between managers from the payer and from the provider sides. The English NHS negotiations focused, informants said, mostly on costs (and waiting times, for one hospital). Over time, trust and goodwill accumulated. The negotiators recognised that they would need each other's help and goodwill in future. A cap-and-collar agreement was often linked with other bargains (e.g. letting a hospital close beds provided that its activity levels stayed above the ‘collar’). In our German site, the SHIs negotiated collectively with each hospital. Negotiations focused on the hospital's DRG points allocation, case mix and the nationally defined growth margin, which together implied an overall number of DRG points and therefore budget. It was possible to enlarge or add, reduce or even remove, care groups by re-allocation within the total number of points, which was how the ‘corridor’ was agreed. Lombardy used two kinds of contract. One, with juridical status, stated the main rules and quality standards that the provider must comply with but the other, annual operational contract had no legal status and this was the one which defined the provider's budget and activity level, within local targets set by the region. These relationships have been described as more like a ‘compact’ than a ‘contract’ ( Powell, 2007 ).

These negotiated arrangements were in all three systems formalised in the sense of being explicitly stated and documented but informal in the sense of being unofficial additions to the laws, decrees or regulations governing the contracts and their juridical status.

## 5. Discussion

Across the three countries, essentially the same managerial workarounds of the DRG system emerged. They had no statutory basis, but were enacted collectively and by explicit negotiation. Insofar as payer–provider negotiations are inherent to a DRG system, the aforementioned workarounds might be regarded as an example of managers extending an official inter-organisational structure and informational resources for additional uses, that is, system planning and cost control. They exploited and stretched managerial discretion. The DRG workarounds did not much alter or supplement what organisational-level or inter-organisational monitoring data the managers received, but payer managers did make additional, non-standard uses of boundary objects (contracts, spreadsheets, etc.) and so widened the possible range of managerial responses to those data. In England and Germany, these and other workarounds were used to develop and support emerging inter-provider care networks for providing integrated care. However, these managerial workarounds did not have all the characteristics that our initial conceptualisation predicted. They added rather than removed warning systems and activity over-rides and increased rather than reduced managerial work. They did not involve alternative diagnostic typologies, nor fudging or re-negotiating diagnostic codes. Neither insufficient resources or time for managerial work, nor its over-complexity, motivated the workarounds reported earlier. Neither did any threat to managers' occupational interests. Given a policy mandate for cost control, the workarounds attenuated what payers foresaw (and sometimes experienced) as perverse consequences of the DRG system, in the absence of official organisational structures or systems for controlling or mitigating these consequences.

These findings have various limitations and caveats. We report DRG workarounds only for three European systems. However, DRG system modifications similar to those we investigated have been reported (or rather, mentioned briefly) in studies of Austria, Greece ( Polyzos *et al.* , 2013 ), Ireland, Portugal, Spain (Catalonia), Sweden, Poland ( Kowalska, 2007 ) and parts of the United States ( Cutler and Ghosh, 2012 ). Other writers report the ‘bundling’ of patient episodes, meaning the workaround described earlier which ‘bundles’ together the payments for the multiple patients in the same DRG group. Others, especially in the United States but also, for example, in the Netherlands ( Bakker *et al.* , 2012 ), use the term ‘bundle’ differently to mean combining all the different DRG payments for one individual patient who receives multiple treatments, perhaps from multiple providers, similar to the NHS ‘year of care’ model described earlier. Although we focused on hospitals, DRG-like systems are increasingly being applied to non-hospital services. In many countries DRGs coexist with non-DRG or pre-DRG systems ( Cots *et al.* , 2011 ). If workarounds emerge from particular local health system settings, our findings may also reflect a specific stage in DRG-system development, perhaps one that will pass. We have reported how payers managed DRGs at inter-organisational level and in light of broad health policy aims, rather than more technical DRG development such as a new ICD, groupers, provider cost accounting systems, ways of converting DRG tariffs into money prices or modelling methods. Neither have we considered how hospital competition may affect DRG workarounds. We selected payers whose DRG systems were well-developed in terms of their managerial systems, extent and intensity of DRG use. If this selection did bias our findings (which is not obvious), the bias would be towards reporting the most extensive and elaborate managerial workarounds in each DRG system. Healthcare is not the only sector that incubates managerial workarounds ( Ledeneva, 2009 ). Synthesising our findings with those from elsewhere would refine the conceptualisations proposed earlier.

## 6. Conclusion: managerial workarounds as diagnostic – and remedial?

Nevertheless, this study adds several things to existing knowledge. One is additional evidence about the internal workings of DRG systems in three health systems, in particular a workaround which is often noticed but seldom deeply examined. So far as we are aware, no other study develops the concept of a ‘managerial workaround', tests it empirically or explains how managerial workarounds occur. In motivation, means and consequences, managerial workarounds differ from most forms of policy–practice gap reported in the implementation literature. Managers' motivation was not to make the DRG system do something other than policymakers intended, as happens when non-managers ‘capture’ a policy or when policy goal displacement ( Abramson, 2009 ) occurs. Neither did the present workarounds arise from managers failing to understand how to enact the DRG policy, nor from policy ambiguity or mis-specification, for DRG systems are clearly specified and highly formalised. Rather the opposite: those making the workarounds intended to achieve policymakers' aims more fully and despite (in this case) some of the incentives arising from the commodified character of DRGs. Their motivation was not primarily symbolic (e.g. external image management, increasing managerial power over clinicians) as institutionalist theory might suggest but concrete and practical (in this case, cost control, case mix and referral planning). It was remedy not resistance.

As means to realise these intentions, the managerial workarounds reported here, like work-process workarounds generally, go beyond passive non-implementation, shortcuts ( Lalley and Malloch, 2010 ) or just exercising discretion *within* the structures that a given policy creates. They extended the structures that DRG policy introduced. Because these extensions were formalised, impersonal and stable, they were capable of becoming normalised ( May and Finch, 2009 ), over time, as permanent inter-organisational structures. These developments were indeed emergent, but from conflicts between policy goals (stimulating hospital activity *versus* cost control) not conflicts between interest groups or conflicting implementation structures for a single policy. Consequently the managerial workarounds ‘decoupled’ organisations' actual work processes only in a specific, restricted way from those organisations' formal structures and external image presentation ( Covaleski *et al.* , 1993 ). Managers' work practices were only decoupled from DRGs' policy-defeating consequences, not from the normative foundations of the DRG system. Hence, the managerial workarounds described here should not necessarily be dismissed (as policy–practice gaps often are) as implementation failures. Rather, they were implementation repairs.

Our findings suggest that a widespread managerial workaround may therefore be a response to policy incoherence. The workarounds that we report appear to be symptomatic of design problems with DRGs, and therefore DRG systems, at a basic level. What motivated the DRG workarounds was the practical incompatibility of two policy objectives. One was DRGs' market conformity. DRGs were designed as a commodified payment system (payment per episode of care) to incentivise providers to increase activity (and on some definitions, ‘efficiency'). The other objective was system-wide cost control. To remove that underlying incompatibility would require policy shifts, but the managerial workarounds themselves may suggest what shifts. The workarounds described earlier produced provider contracts that retained the informational strengths of DRGs, made providers' expected incomes and case mix transparent and uniformly defined across providers, while also defining the payers' care costs prospectively and constituting a means of cost control for both payers and providers. The managerial workaround converted DRG payments into a specific kind of flexible, weakly incentivised global (‘block’) contract system, a hybrid system for assembling flexible global budgets from estimates of patient-level activity and DRG payments.

Health policy researchers who discover pervasive, non-trivial managerial workarounds might therefore consider them a prompt to investigate further whether that discovery is symptomatic, in turn, of inconsistent policy objectives, or of a conflict between those objectives and the organisational structures intended to realise them, but also whether the workarounds themselves may contain solutions to these problems.
